# An Apparent Case of Salivation

**Published:** 1896-09

**Authors:** C. W. Stainton

**Affiliations:** Buffalo, N. Y.


### AN APPARENT CASE OF SALIVATION.

BY C. W. STAINTON, D.D.S., BUFFALO, N. Y.

The recent sickness and death of a neighbor of seventy-five years furnishes a case of interest from both a medical and a dental stand-point.

In the nearly score of years I have known her she has never been strong. Her appetite was very delicate, and but few things could be tolerated, and food in very limited quantity could be taken at once. During the latter part of the past winter she had not been as well as usual, and one of our brightest and most progressive medical men was called to attend her.

He found her entirely free from cardiac, kidney, or hepatic complications; she seemed to be suffering solely from lack of nutrition, and efforts were directed towards obviating this difficulty. A few days' trial seemed to show that progress in this direction was not likely to be encouraging, from one-half to one teaspoonful of beef-tea being the limit. Any larger quantity caused considerable distress, and was soon ejected.

The secretion of saliva was abnormally large, and this constantly swallowed saliva was ejected towards the close of each day. After some time spent in unsatisfactory trial the physician in charge called in consultation a professional friend, who has made gastric troubles a more especial study than any one else in our city.

The attending physician had for some days entertained the suspicion that an old, red-rubber, upper set of teeth, which had been worn for twenty-five years, might be the source of the trouble.

The appearance of red, inflamed patches of the mucous membrane under that ill-fitting plate (a condition well understood by dentists as due more to mechanical irritations from ill-fitting, loose-shifting dentures than anything else) and the increased flow of saliva pointed in this direction.

The consulting physician coincided in this view. A suspicion was entertained by both that the pancreas was not performing its full duty. If this were true it would in part explain the conditions present. The set of teeth was removed from the patient's mouth and turned over to the professor of chemistry in the University of Buffalo. He took showings from the plate, applied the proper tests, and found mercurial reaction.

The suspicion that mercurial salivation was the chief trouble now seemed proved. Death of the patient subsequently occurred.

An autopsy revealed a stomach very much reduced in size, as though a surgical operation had removed all the depending portion, so that its appearance resembled a section of the large intestine.

This may offer an explanation of the small quantity of food tolerated at one time and also the periodical rejection of the swallowed saliva.

The liver was reduced in size considerably, but was secreting bile; the duct, very small, was open, and bile found in it as well as in the duodenum. The gall-bladder was full of gall-stones. The pancreas, like all the viscera, was much reduced in size, its structure considerably changed, the connective tissue unusually abundant, and the pancreatic duct *entirely closed.* No pancreatic juice could have been poured into the duodenum for a long time past.

This discovery put a new face on the matter. It was easy now to understand the increased flow of saliva. One of the digestive glands being practically obliterated, a vicarious effort had been made on the part of others to make good the loss.

See what proofs—strong as Holy Writ—in the hands of some theorists we have here to prove a case of salivation from wearing a red rubber plate. 1, the sore patches of mucous membrane; 2, the incessant flow of saliva; 3, the gradual wasting away of the patient; 4, the mercurial test.

The attending physician reported to me that the chemist found free mercury. This was an error. The test was a very delicate one for any preparations or forms of mercury. No free mercury was found.

It is a matter of regret that since the days of Wildman nothing has been added to our literature on rubber and its composition.[1]

---

[1] Investigation of this question was very exhaustively made by the Pennsylvania Association of Dental Surgeons of Philadelphia about twenty-five years ago. That society appointed a committee, consisting of Drs. Wildman, Buckingham, and Truman, to examine into the subject. The mechanical, chemical, and microscopical were given to each in the order named. The report, in substance, demonstrated that the amount of free mercury present in vulcanized plates was infinitesimal, and on the polished surfaces nothing could be found. The writer of this, in numerous sections examined, found in two or three instances exceedingly minute globules under high powers of the microscope; but so thoroughly embedded were these in the impervious rubber that the possibility of causing local disturbance could not be entertained.

The cause of mucous irritation is no doubt due to uncleanliness on the part of patients and the presence of micro-organisms on the plate, as shown by Dr.

Some careful, patient, chemical, microscopical student has a large and important field awaiting him in this direction.

